# Cross-sectional association between plasma aldosterone concentration and cognitive performance by mini-mental state examination in community dwellers

**DOI:** 10.3389/fnut.2025.1519644

**Published:** 2025-02-06

**Authors:** Aketilieke Nusufujiang, Mulalibieke Heizhati, Nanfang Li, Ling Yao, Wenbo Yang, Hui Wang, Mei Li, Lin Gan, Adalaiti Maitituersun, Miaomiao Liu, Qiaolifanayi Nuermaimaiti, Li Cai, Xiayire Aierken, Xiufang Li, Qin Luo, Jing Hong

**Affiliations:** Hypertension Center of People’s Hospital of Xinjiang Uygur Autonomous Region, Xinjiang Hypertension Institute, NHC Key Laboratory of Hypertension Clinical Research, Key Laboratory of Xinjiang Uygur Autonomous Region “Hypertension Research Laboratory”, Xinjiang Clinical Medical Research Center for Hypertension (Cardio-Cerebrovascular) Diseases, Ürümqi, China

**Keywords:** aldosterone, cognitive function, plasma aldosterone concentration, cognition, cognitive performance

## Abstract

**Background:**

Aldosterone is the effector hormone in the renin angiotensin aldosterone system and existing data suggest aldosterone affect cognitive function. However, the relationship between plasma aldosterone concentration (PAC) and cognitive performance remains unexplored in community dwellers. Therefore, we aimed to explore whether PAC is associated with cognitive performance in this population.

**Methods:**

We cross-sectionally enrolled adults using multistage random sampling from Emin, China in 2019. Participants underwent questionnaires and data collection. Cognitive status was assessed using mini-mental state examination (MMSE) questionnaire. Multi-variable linear and logistic regression were used to explore the association between log PAC and log MMSE score, and between tertiled PAC (the higher PAC as the exposure) and low cognitive performance, respectively, in total, apparently healthy and diseased participants. Subgroup analyses also were performed by age, gender, BMI, living region, ethnicity and education attainment status.

**Results:**

27,707 subjects were included, of whom, 12,862 were apparently healthy and 14,845 had disease. Log-PAC was positively associated with log-MMSE score in the multivariable linear regression in the total (*B* = 0.01, 95%CI: 0–0.01, *p* < 0.001), apparently healthy (*B* = 0.01, 95%CI: 0–0.01, *p* = 0.007) participants, and the diseased without taking medicine (*B* = 0.01, 95%CI: 0.01–0.02, *p* = 0.004) participants. In logistic regression, the highest third tertile of PAC group showed significantly lower odds for the presence of low cognitive performance in total (OR = 0.83, 95%CI: 0.73–0.93, *p* = 0.002) and diseased without taking medicine participants (OR = 0.70, 95%CI: 0.57–0.86, *p* < 0.001). Various sub-group analysis showed largely consistent results with the main analysis.

**Conclusion:**

There was a positive correlation between plasma aldosterone and cognitive functions in community dwellers, whereas further studies are need when considering the cross-sectional nature of the current study.

## Introduction

Cognition refers to the process by which people acquire knowledge or apply knowledge, or the process of information processing, including feeling, perception, memory, thinking, imagination and language. Cognitive ability refers to the ability of the human brain information processing, storage and extraction, which we usually speak of intelligence, such as observation, memory, imagination, etc. Cognitive function is particularly important, especially as the population ages, because it determines the maintenance of our independence, the performance of everyday activities, and the quality of life. Several modifiable risk factors for cognitive decline have been identified (1). For example, cardiovascular disease (CVD), hypertension, inflammation, obesity, smoking, and alcohol abuse have been associated with increased risk of cognitive decline and incidence of dementia (2–4). A growing body of research has indicated that aldosterone, a pivotal downstream factor within the renin-angiotensin-aldosterone system (RAAS), possesses the capacity to enhance the survival rate of cochlear nerve cells and impede the progression of the apoptotic pathway. These neuroprotective mechanisms may extend to other parts of the nervous system, thereby protecting cognitive function during aging (5).

The mineralocorticoid receptor (MR) is imperative in the regulation of the stress response, neuroendocrine function and cognitive function, and is involved in the regulation of the integrity and stability of neural networks. Aldosterone exerts its effects on neuronal function, synaptic plasticity, cognitive function and emotion regulation by binding to MR Receptors, which are widely distributed in the brain. The distribution of MR Receptors is particularly pronounced in the hippocampus and prefrontal cortex, regions that are well-established to be integral to cognitive function (6). Furthermore, the over-expression of forebrain MR has been shown to enhance memory performance and reduce neuronal loss during cerebral ischemia in mice (7). In a study on the effect and mechanism of mineralocorticoid receptor (MR) activation on postoperative hippocampal neurogenesis and cognitive function, aldosterone induced the phosphorylation of Akt and GSK-3b, and promotes the proliferation of hippocampal neural stem cells and improves cognitive dysfunction in aged mice after surgery (8). In another animal study, aldosterone may promote the proliferation and survival of newly-generated granule cells in the dentate gyrus of adrenalectomized rat (9). Some animal studies have shown that overexpression of MR enhances memory and stimulating MR enhances longterm potentiation, while decreased expression of MR in the hippocampus can lead to spatial memory impairment and working memory deficits (7, 10).

However, the role of PAC in cognition has not been fully explored in humans and there are inconsistencies among the study results. In a study of 138 patients with white matter lesions, plasma aldosterone concentrations were associated with white matter lesions in patients with primary aldosteronism (11). In another study from our center of 547 hypertensive patients with white matter lesions, higher PAC, especially PAC > 17.26 ng/dL, increased the risk of white matter lesions, and PAC was positively correlated with white matter lesions (12). However, in a prospective cohort study, reduced physiological hydration status was associated with greater reductions in global cognitive function over a 2-year period in 1957 older adults (13).A previous study reported a negative effect of aldosterone on cognition among hypertensives, enrolled 68 patients with essential hypertension. Mineral corticoid receptor antagonists, including spironolactone and eplerenone, increased MMSE score in seven patients with hypertension, but not in the controls (14). Moreover, in a clinical trial, individuals that were older adults (n = 47; mean age = 71 years) with the highest aldosterone levels at baseline showed the greatest improvement in executive functioning after 12 months of BP lowering treatment(<140/90 mm Hg), Higher levels of aldosterone may be associated with decreased cerebrovascular function in hypertension. Rimmele et al. (15) showed that MR blockade can impair the memory function of young healthy men. Stimulation of the MR has been previously found to improve memory in young and elderly healthy individuals, as well as depressed patients (16, 17).

Aldosterone is a hormone that preserves sodium, water and discharges potassium. Decreased concentration of aldosterone leads to decreased water retention ability and decreased cognitive function, indicating that there is a positive correlation between aldosterone and cognitive function. A cross-sectional study in the community population in recent years found that there was an independent association between higher 24-h urinary sodium to potassium ratio and mild cognitive impairment (18), which may indirectly indicate that aldosterone may be related to cognitive function. The role of aldosterone in brain health warrants further investigation in a larger trial (19). In addition, it is not difficult to observe that above studies were mainly carried out in clinical patient population.

Therefore, it is necessary to analyze the relationship between circulating aldosterone and cognitive function in humans, in order to understand the effect of aldosterone on cognitive function. This study uses cross-sectional study in community dwellers, to investigate the relationship between the PAC and cognitive function in community dwellers.

## Methods

### Study population

In this cross-sectional study, we used multi-stage stratified sampling method to enroll study population aged ≥18 years, as in our previous studies (18). At the first stage,the whole county was divided into three regions as urban, agricultural and stock-raising regions. At the second stage, two townships were randomly selected in each region using simple random sampling. At the third stage, two villages were randomly selected as survey villages in each of the extracted townships. In the final stage of sampling, a given number of participants from each site were selected from communities or villages using lists compiled from local government registers of households.

Inclusion criteria encompassed: (1) local inhabitants aged ≥18 years; (2) residing at current address for ≥6 months; (3) agreeing to participate and sign an informed consent form; (4) participants with complete blood samples, aldosterone concentrations, and MMSE data. Exclusion criteria included: inability to cooperate with investigators due to hearing impairment, communication impairment, intellectual disability, and mental problems.

### Data collection

### Questionnaire, physical examination, and biochemical examination

Population health behavior questionnaires and physical examinations were conducted using onsite surveys to collect detailed information from all participants via a face-to-face interview by trained investigators, which included demographic characteristics (name, gender, age, ethnicity and current address), socioeconomic status (occupation and educational status), lifestyle risk factors (cigarette consumption and alcohol intake), individual and family medical history(hypertension, diabetes, coronary heart disease, and stroke). Physical examination included measurements of height, body weight, waist circumference (WC) and blood pressure (BP). Each participant completed questionnaires on face-to-face interview including MMSE, global physical activity questionnaire (GPAQ), Pittsburgh Sleep Quality Index (PSQI), Zung’s Self-Rating anxiety and depression scale (SAS and SDS) questionnaires, and No-SAS scale. Laboratory examination included measurement of fasting blood glucose, lipid profiles, transaminases and creatinine.

### Measurement of BP, height, weight, and waist circumference

BP was presented as the mean of three measurements using an Omron HEM-1000 electronic sphygmomanometer (20). All participants were advised to avoid cigarette smoking, alcohol, caffeinated beverages, tea and exercise for at least 30 min prior to measurements. Three BP measurements were taken, after a rest of at least 5 min, from the unclothed right arm of the person in a sitting position at an interval of at least 1 min. Body weight, height and WC were measured using standard methods (21). Height and weight were measured to the nearest 0·1 cm and 0·1 kg, respectively, with the participants in lightweight clothing and without shoes. WC was measured at the midpoint between the lower rib and upper margin of the iliac crest to the nearest 0·1 cm at the end of a normal expiration. BMI was calculated by dividing weight by height-squared (kg/m2).

### Laboratory measurements

Venous blood samples were obtained by the trained nurses in the morning after overnight fasting. After resting at room temperature for 30 min, the upper layer of serum was centrifuged at 3000 RPM for 20 min at 4 ° C and placed in a 2mlEP tube. The collected EP tubes were immediately stored in a portable refrigerator at a temperature as low as-20 ° C, and then transferred to the Hypertension Center of Xinjiang Uygur Autonomous Region People’s Hospital (located in Urumqi, Xinjiang, a distance of 500 km) and stored in a refrigerator at a temperature as low as-80 ° C until the measurement in 2021. Aldosterone concentrations before test, blood samples were transferred to - 20°C refrigerator, and returned to the greenhouse before use. The plasma aldosterone were measured by the staff blinded to the aim and design of the study. PAC was measured using radioimmunoassay (DSL-8600 ACTIVE® Aldosterone Coated Tube Radioimmunoassay Kit; Diagnostic Systems Laboratories, Webster, TX, USA) with the intra-and inter-assay coefficients of variation of 5.6 and 8.5% in both data. The details of the measurements are described in previous studies from our center (22, 23).

### Assessment of cognitive status

Trained investigators evaluated cognitive status with MMSE (24). The scale assesses the following five aspects: orientation, immediate memory, attention and calculation, transient recall, and language expression. The total score of the scale ranges from 0 to 30 points, and the assessment time is about 5–10 min. The cognitive decline assessed by MMSE was related to the years of education: low cognitive performance is defined as MMSE score < 17, < 20 and < 24 for subjects with no formal education, 1–6 years of and with ≥7 years of education, respectively (25).

Hypertension is defined as systolic blood pressure (SBP) ≥140 mm Hg, and/or diastolic blood pressure (DBP) ≥90 mmHg, and/or use of antihypertensive medicine within 2 weeks, based on the 2018 Chinese Hypertension Guideline (26). Dyslipidemia is defined as having TC ≥ 6.22 mmol/L, LDL-C ≥ 4.14 mmol/L, HDL-C ≤ 1.04 mmol/L, TG levels ≥2.26 mmol/L, or self-reported use of lipid-lowering medications, in accordance with the 2016 Chinese Adult Dyslipidemia Prevention Guideline (27). Type 2 diabetes, as defined by the American Diabetes Association (28), is characterized by FPG levels ≥7.0 mmol/L and/or current use of antidiabetic drugs.

### Statistical analysis

Current study is a post-hoc analysis of the whole data collected in Emin in 2019. Participants were grouped by tertile of PAC as T1 (the lowest tertile of PAC, <11.54 ng/dL), T2 (the second tertile of PAC 11.54–18.15 ng/dL) and T3 (the hihgest tertile of PAC >18.15 ng/dL) groups. Data analysis for the association between PAC and MMSE or low cognitive performance were performed in total participants, apparently healthy and diseased participants without taking medication.

Apparent diseases included: (1) hypertension; (2) self-reported coronary heart disease, myocardial infarction, or coronary angioplasty or stent angioplasty, coronary artery bypass, and or stroke; (3) Dyslipidemia; (4) Type 2 diabetes; (5) eGFR <60 mL/(min·1.73㎡) and ALT or AST > 3 times the normal value.

Students’ *t*-test was used to assess between-group differences in continuous variables if normally distributed; otherwise, nonparametric (Mann– Whitney U) test was applied. X2-test was used to assess between-group differences of categorical variables. P for trend was calculated by Kruskal–Wallis H test and X2 trend test for ordinal variables.

Multi-variable linear and logistic regression were used to explore the association between log PAC and log MMSE score, and between PAC (the lowest tertile PAC as the referrence and the higher second and third tertile of PAC as the exposure) and low cognitive performance, respectively in total, apparently healthy and diseased participants without taking medication. In addition, subgroup analyses were performed in different age, gender, BMI, living region, ethnicity and education attainment status.

Results were expressed as regression coefficients (B value) and as odds ratios (ORs) and 95% confidence intervals (95% CI). Before creating regression models, independent variables significantly relevant to MMSE score were selected using uni-variate linear regression (Supplementary Table 3). If P was <0.1, these variables are included. Tolerance and variance inflation factor (VIF) were examined to identify multicollinearity and multicollinearity is a concern if VIF is >10 and the tolerance is <0.10 (29).

SPSS 27.0 software was applied for statistical analysis of the data. The test level was set at *ɑ* = 0.05 and a two-sided *p* value <0.05 was considered statistically different.

## Results

**Figure 1:**
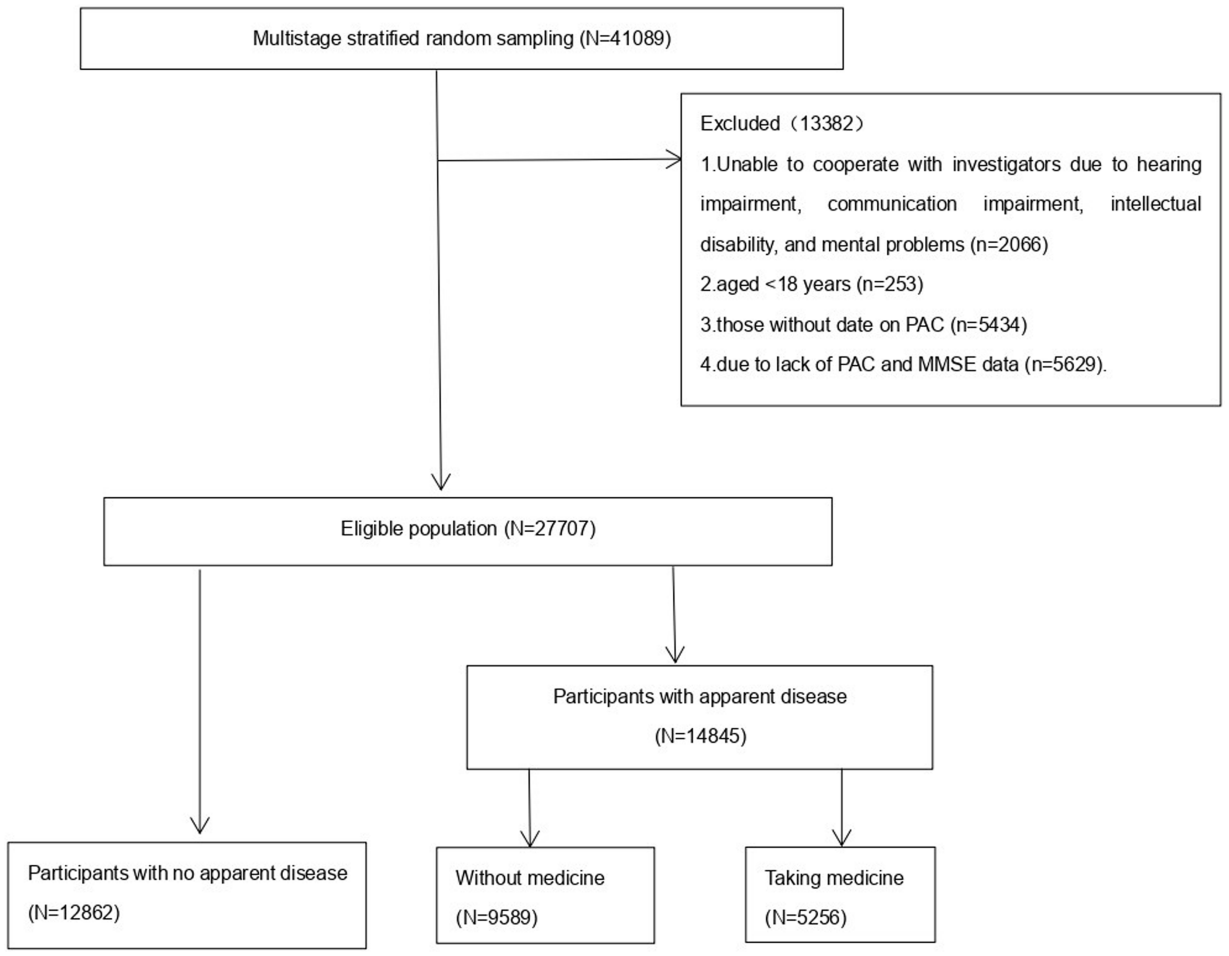
Flowchart for study population.

As shown in the flowchart (Figure 1), 27,707 subjects with complete data on MMSE and PAC were included, of whom, 12,862 were apparently healthy and 14,845 had disease. Among the diseased population, 9,589 were not taking any medication and 5,256 were taking antihypertensive or antidiabetic or antilipemic drugs.

### Baseline population characteristics

As shown in Table 1, average age of participants were 47 years and 53.4% were women. Participants in the T3 group of PAC were more likely to be women, young, Han, urban dwellers, and less likely to smoke, compared to those in the T1 group.

**Table 1:** Characteristics of study participants by total and by the tertile of PAC.

	Total	<11.54 ng/dL	11.54–18.15 ng/dL	>18.15 ng/dL	*P*
*N*	27,707	9,235	9,235	9,237	
Age (years)	47 (37, 56)	48 (37, 57)	48 (38, 57)	46 (35, 54)	<0.001
≤ 44 years	11,719 (42.3)	3,740 (40.5)	3,629 (39.3)	4,350 (47.1)	
45–59 years	10,837 (39.1)	3,604 (39.0)	3,772 (40.8)	3,461 (37.5)	
≥60 years	5,151 (18.6)	1891 (20.5)	1834 (19.9)	1,426 (15.4)	
Women (*n*, %)	14,801 (53.4)	4,154 (28.1)	5,058 (34.2)	5,589 (37.8)	<0.001
BMI (kg/m2)	25.4 (22.8, 28.3)	25.4 (22.8, 28.4)	25.6 (23.0, 28.4)	25.3 (22.6, 28.1)	<0.001
BMI 25–30	10,773 (38.9)	3,542 (38.4)	3,683 (39.9)	3,548 (38.5)	<0.001
BMI ≥30	4,228 (15.3)	1,462 (15.8)	1,450 (15.7)	1,316 (14.3)	<0.001
WC (cm)	86.6 (78.2, 95.0)	87 (79, 95.3)	87 (79, 95)	86 (77.3, 94.5)	<0.001
Obesity (*n*, %)	13,250 (47.8)	4,411 (33.3)	4,498 (33.9)	4,341 (32.8)	0.067
Han (*n*, %)	13,525 (48.8)	3,584 (26.5)	4,612 (34.1)	5,329 (39.4)	<0.001
Ethnic minority (*n*, %)	14,182 (51.1)	5,651 (39.8)	4,623 (32.6)	3,908 (27.6)	
Education (*n*, %) ≤ Primary	8,414 (30.3)	3,285 (39.0)	3,015 (35.8)	2,114 (25.1)	<0.001
Junior high	10,635 (38.3)	3,750 (35.3)	3,690 (34.7)	3,195 (30.0)	
≥Senior high	8,657 (31.2)	2,200 (25.4)	2,530 (29.2)	3,927 (45.4)	
Region (*n*, %) urban	10,019 (36.1)	2,737 (29.6)	3,125 (33.8)	4,157 (45.0)	<0.001
rural	17,688 (63.8)	6,498 (70.4)	6,110 (66.2)	5,080 (55.0)	
Occupation (*n*, %) physical	17,012 (61.3)	6,044 (35.5)	5,998 (35.3)	4,970 (29.2)	<0.001
Marriage (*n*, %) married	22,787 (82.3)	7,541 (81.7)	7,710 (83.5)	7,536 (81.6)	<0.001
Cigarette use (*n*, %)	7,254 (26.1)	3,100 (42.7)	2,284 (31.5)	1870 (25.8)	<0.001
Alcohol use (*n*, %)	9,209 (33.2)	3,283 (35.6)	2,959 (32.1)	2,967 (32.2)	<0.001
SBP (mmHg)	123 (111, 138)	125 (113, 140)	123 (111, 138)	120 (110, 135)	<0.001
DBP (mmHg)	79 (70, 88)	80 (70, 88)	79 (70, 88)	78 (70, 87)	<0.001
Total MET minutes	3,600 (1,680, 9,240)	5,040 (1,680, 10,080)	4,200 (1,680, 10,080)	3,360 (1,008, 7,800)	0.004
PSQI score	4 (2, 7)	4 (2, 7)	4 (2, 7)	4 (2, 7)	0.963
NoSAS score	5 (2, 7)	5 (2, 8)	5 (2, 7)	4 (2, 7)	<0.001
SDS score	30 (26, 36)	30 (26, 35)	30 (26, 35)	30 (28, 36)	0.01
SAS score	30 (26, 35)	30 (26, 35)	30 (26, 35)	30 (26, 36)	0.03
Hypertension (*n*, %)	9,825 (35.5)	3,472 (37.6)	3,318 (35.9)	3,035 (32.9)	0.270
CVD (*n*, %)	1,168 (4.2)	355 (30.4)	385 (33.0)	428 (36.6)	0.034
Diabetes (*n*, %)	3,029 (10.9)	990 (32.7)	1,072 (35.4)	967 (31.9)	0.054
Dyslipidemia (*n*, %)	8,390 (30.2)	2,784 (33.2)	2,863 (34.1)	2,743 (32.7)	0.257
Abnormal liver function (*n*, %)	123 (0.4)	38 (30.9)	41 (33.3)	44 (35.8)	0.803
Renal insufficiency (*n*, %)	1,152 (4.4)	333 (3.6)	403 (4.4)	416 (4.5)	0.003
eGFR	100.3 (83.7, 112.5)	100.4 (85.7, 111.9)	98.7 (81.6, 110.6)	101.8 (83.7, 114.9)	<0.001
TC (mg/dl)	4.70 (4.00, 5.50)	4.72 (4.03, 5.60)	4.77 (4.00, 5.54)	4.60 (3.90, 5.40)	<0.001
Triglyceride (mg/dl)	1.20 (0.85, 1.78)	1.20 (0.90, 1.75)	1.21 (0.90, 1.80)	1.20 (0.80, 1.70)	<0.001
FBG (mmol/l)	5.30 (4.82, 5.87)	5.34 (4.90, 5.91)	5.32 (4.83, 5.91)	5.21 (4.72, 5.78)	<0.001
PAC (ng/dl)	14.44 (10.28, 20.60)	8.98 (7.59, 10.28)	14.44 (12.91, 16.16)	23.95 (20.60, 29.82)	

Data are presented as mean ± SD/median, interval/*n* (%); BMI, Body mass index; WC, waist circumference; SBP, systolic blood pressure; DBP, diastolic blood pressure; PSQI score, Pittsburgh sleep quality index; SDS score, self rating depression score; SAS score, self rating anxiety score; CVD, cardiovascular diseases; eGFR, estimated glomerular filtration rate; TC, total cholesterol; FBG, fasting blood glucose; PAC, plasma aldosterone concentration.

Before creating regression models, independent variables significantly relevant to MMSE score were selected using uni-variate linear regression (Supplementary Table 3). If *P* was <0.1, these variables are included. Tolerance and variance inflation factor (VIF) were examined to identify multicollinearity and multicollinearity is a concern if VIF is >10 and the tolerance is <0.10 (29). Model 1 was adjusted for age, gender, BMI, waist circumference, ethnicity, education, region, occupation, and marital status. Model 2, adjusted for model 1 + smokers, drinkers, SBP, DBP, PSQI score, MET Minutes, NoSAS score, SDS score, SAS score, total cholesterol, triglyceride, glutamic oxalacetic transaminase,and fasting blood glucose.

### Linear and logistic regression models

As shown in Tables 2–4, the multivariable linear regression analysis showed that log-PAC were positively associated with log-MMSE score in total (*B* = 0.01, 95%CI: 0–0.01, *p* < 0.001), in apparently healthy adults (*B* = 0.01, 95%CI: 0–0.01, *p* = 0.007), and in the diseased (*B* = 0.01, 95%CI: 0.01–0.02, *p* = 0.004) participants without taking medicine.

**Table 2:** Linear and logistic regression analysis for the association of PAC with MMSE in total and stratified participants by sex, age (B/OR, 95%CI, P).

	Crude model	Model 1	Model 2
Linear regression analysis			
Total participants	0.04 (0.03, 0.04), <0.001	0.01 (0, 0.01), 0.002	0.01 (0, 0.01), <0.001
Stratification by sex			
Men	0.04 (0.03, 0.04), <0.001	0.01 (0, 0.01), 0.005	0.01 (0, 0.01), 0.009
Women	0.05 (0.04, 0.06), <0.001	0.01 (0, 0.01), 0.185	0.01 (0, 0.01), 0.086
Stratification by age			
<45 years	0.02 (0.02, 0.03), <0.001	0.01 (0, 0.01), 0.035	0.01 (0, 0.01), 0.047
45–59	0.04 (0.03, 0.05), <0.001	0.01 (0.01, 0.02), 0.002	0.01 (0.01, 0.02), <0.001
≥60 years	0.02 (0.01, 0.04), <0.001	0 (−0.01, 0.01), 0.994	0 (−0.01, 0.01), 0.972
Logistic regression			
Total participants			
T2 vs. T1	0.94 (0.84, 1.04), 0.203	0.97 (0.87, 1.08), 0.530	0.95 (0.85, 1.06), 0.352
T3 vs. T1	0.81 (0.72, 0.90), 0.805	0.86 (0.77, 0.97), 0.012	0.83 (0.73, 0.93), 0.002
Stratification by sex			
Men			
T2 vs. T1	0.89 (0.75, 1.05), 0.162	0.97 (0.82, 1.15), 0.725	0.94 (0.79, 1.13), 0.515
T3 vs. T1	0.81 (0.68, 0.97), 0.020	0.78 (0.64, 0.95), 0.011	0.75 (0.62, 0.92), 0.006
Women			
T2 vs. T1	0.90 (0.78, 1.03), 0.115	0.97 (0.84, 1.11), 0.621	0.96 (0.83, 1.10), 0.533
T3 vs. T1	0.72 (0.63, 0.83), <0.001	0.90 (0.78, 1.05), 0.176	0.87 (0.75, 1.01), 0.075
Stratification by age			
<45 years			
T2 vs. T1	0.89 (0.72, 1.09), 0.254	0.87 (0.70, 1.07), 0.187	0.83 (0.67, 1.04), 0.106
T3 vs. T1	0.90 (0.74, 1.10), 0.290	0.81 (0.65, 1.00), 0.047	0.79 (0.64, 0.99), 0.040
45–59 years			
T2 vs. T1	0.93 (0.79, 1.09), 0.370	0.95 (0.81, 1.12), 0.550	0.95 (0.80, 1.13), 0.550
T3 vs. T1	0.76 (0.64, 0.90), 0.002	0.78 (0.65, 0.93), 0.007	0.73 (0.60, 0.89), 0.001
≥60 years			
T2 vs. T1	0.97 (0.81, 1.17), 0.779	1.02 (0.84, 1.23), 0.842	1.00 (0.82, 1.22), 0.966
T3 vs. T1	0.97 (0.80, 1.19), 0.792	1.02 (0.82, 1.25), 0.887	0.96 (0.77, 1.20), 0.720

Model 1 was adjusted for age, gender, BMI, waist circumference, ethnicity, education, region, occupation, and marital status. Model 2, adjusted for model 1 + smokers, drinkers, SBP, DBP, PSQI score, MET Minutes, NoSAS score, SDS score, SAS score, total cholesterol, triglyceride, glutamic oxalacetic transaminase, and fasting blood glucose.

**Table 3:** Linear and logistic regression analysis for the association of PAC with MMSE in total and stratified participants by sex, age (B/OR, 95%CI, P) in people with no apparent disease.

	Crude model	Model 1	Model 2
Linear regression analysis			
Total participants	0.03 (0.03, 0.04), <0.001	0.01 (0, 0.01), 0.022	0.01 (0, 0.01), 0.007
Stratification by sex			
Men	0.02 (0.01, 0.03), <0.001	0 (−0.01, 0.01), 0.749	0.01 (−0.01, 0.01), 0.482
Women	0.04 (0.03, 0.05), <0.001	0.01 (0, 0.01), 0.036	0.01 (0.01, 0.02), 0.02
Stratification by age			
<45 years	0.02 (0.01, 0.03), <0.001	0.01 (−0.01, 0.01), 0.113	0.01 (0, 0.01), 0.068
45–59	0.03 (0.02, 0.04), <0.001	0.01 (0, 0.02), 0.186	0.01 (−0.01, 0.02), 0.146
≥60 years	0.02 (−0.01, 0.05), 0.133	0.01 (−0.02, 0.04), 0.541	0.01 (−0.02, 0.04), 0.594
Logistic regression			
Total participants			
T2 vs. T1	0.97 (0.82, 1.15), 0.723	0.97 (0.81, 1.16), 0.770	0.94 (0.78, 1.13), 0.512
T3 vs. T1	0.86 (0.72, 1.03), 0.102	0.86 (0.71, 1.05), 0.131	0.83 (0.68, 1.02), 0.072
Stratification by sex			
Men			
T2 vs. T1	0.93 (0.69, 1.24), 0.600	0.97 (0.72, 1.32), 0.849	0.95 (0.70, 1.31), 0.762
T3 vs. T1	0.94 (0.69, 1.30), 0.723	0.91 (0.65, 1.28), 0.597	0.88 (0.62, 1.26), 0.487
Women			
T2 vs. T1	0.93 (0.75, 1.16), 0.515	0.97 (0.78, 1.22), 0.805	0.95 (0.75, 1.20), 0.658
T3 vs. T1	0.75 (0.60, 0.93), 0.010	0.86 (0.68, 1.081), 0.192	0.84 (0.65, 1.07), 0.147
Stratification by age			
<45 years			
T2 vs. T1	0.95 (0.74, 1.23), 0.702	0.92 (0.71, 1.19), 0.512	0.87 (0.67, 1.14), 0.303
T3 vs. T1	0.94 (0.74, 1.20), 0.635	0.85 (0.65, 1.10), 0.208	0.81 (0.62, 1.06), 0.125
45–59 years			
T2 vs. T1	1.05 (0.79, 1.39), 0.749	1.03 (0.77, 1.38), 0.827	1.05 (0.78, 1.43), 0.736
T3 vs. T1	0.93 (0.68, 1.25), 0.615	0.89 (0.65, 1.23), 0.492	0.87 (0.62, 1.22), 0.417
≥60 years			
T2 vs. T1	1.047 (0.79, 1.39), 0.749	0.92 (0.57, 1.48), 0.726	0.87 (0.52, 1.47), 0.605
T3 vs. T1	0.93 (0.68, 1.25), 0.615	0.83 (0.46, 1.49), 0.523	0.93 (0.49, 1.78), 0.833

Model 1 was adjusted for age, gender, BMI, waist circumference, ethnicity, education, region, occupation, and marital status. Model 2, adjusted for model 1 + smokers, drinkers, SBP, DBP, PSQI score, MET Minutes, NoSAS score, SDS score, SAS score, total cholesterol, triglyceride, glutamic oxalacetic transaminase,and fasting blood glucose.

**Table 4:** Linear and logistic regression analysis for the association of PAC with MMSE in total and stratified participants by sex, age (B/OR, 95%CI, P) in people with apparent disease without taking medicine.

	Crude model	Model 1	Model 2
Linear regression analysis			
Total participants	0.04 (0.04, 0.05), <0.001	0.01 (0.01, 0.02), 0.002	0.01 (0.01, 0.02), 0.004
Stratification by sex			
Men	0.05 (0.04, 0.06), <0.001	0.01 (0.01, 0.02), 0.001	0.02 (0.01, 0.02), 0.003
Women	0.06 (0.05, 0.07), <0.001	0.01 (−0.01, 0.02), 0.319	0.01 (−0.01, 0.02), 0.355
Stratification by age			
<45 years	0.03 (0.02, 0.04), <0.001	0.01 (−0.01, 0.02), 0.234	0.01 (−0.01, 0.02), 0.272
45–59	0.05 (0.04, 0.06), <0.001	0.02 (0.01, 0.03), <0.001	0.02 (0.01, 0.03), 0.002
≥60 years	0.03 (0.01, 0.05), 0.018	0.01 (−0.02, 0.03), 0.434	0.01 (−0.01, 0.03), 0.350
Logistic regression			
Total participants			
T2 vs. T1	0.75 (0.62, 0.89), 0.001	0.75 (0.62, 0.91), 0.003	0.73 (0.60, 0.89), 0.002
T3 vs. T1	0.70 (0.58, 0.84),<0.001	0.72 (0.59, 0.88), 0.001	0.70 (0.57, 0.86), <0.001
Stratification by sex			
Men			
T2 vs. T1	0.81 (0.63, 1.05), 0.114	0.91 (0.69, 1.19), 0.482	0.88 (0.67, 1.16), 0.373
T3 vs. T1	0.65 (0.49, 0.86), 0.002	0.62 (0.46, 0.84), 0.002	0.58 (0.42, 0.80), <0.001
Women			
T2 vs. T1	0.61 (0.47, 0.79), <0.001	0.64 (0.49, 0.83), <0.001	0.62 (0.48, 0.82), <0.001
T3 vs. T1	0.64 (0.50, 0.82), <0.001	0.78 (0.60, 1.02), 0.071	0.79 (0.60, 1.04), 0.092
Stratification by age			
<45 years			
T2 vs. T1	0.82 (0.55, 1.22), 0.318	0.82 (0.54, 1.23), 0.326	0.81 (0.53, 1.23), 0.321
T3 vs. T1	0.78 (0.53, 1.15), 0.205	0.70 (0.46, 1.05), 0.081	0.74 (0.49, 1.13), 0.162
45–59 years			
T2 vs. T1	0.88 (0.68, 1.13), 0.313	0.90 (0.69, 1.18), 0.444	0.91 (0.69, 1.19), 0.481
T3 vs. T1	0.64 (0.49, 0.85), 0.002	0.64 (0.48, 0.87), 0.004	0.59 (0.43, 0.81), 0.001
≥60 years			
T2 vs. T1	0.50 (0.35, 0.71), <0.001	0.49 (0.34, 0.70), <0.001	0.47 (0.32, 0.69), <0.001
T3 vs. T1	0.89 (0.64, 1.24), 0.506	0.88 (0.62, 1.24), 0.452	0.82 (0.57, 1.19), 0.297

Model 1 was adjusted for age, gender, BMI, waist circumference, ethnicity, education, region, occupation, and marital status. Model 2, adjusted for model 1 + smokers, drinkers, SBP, DBP, PSQI score, MET Minutes, NoSAS score, SDS score, SAS score, total cholesterol, triglyceride, glutamic oxalacetic transaminase,and fasting blood glucose.

In logistic regression, the highest third tertile of PAC group showed significantly lower odds for the presence of low cognitive performance in total (OR = 0.83, 95%CI: 0.73–0.93, *p* = 0.002) as in Table 2, diseased without taking medicine participants (OR = 0.70, 95%CI: 0.57–0.86, *p* < 0.001) as in Table 4. No significant association was observed between the two in apparently healthy adults (OR = 0.83, 95%CI: 0.68–1.02, *p* = 0.072) in logistic regression.

The prevalence of low cognitive performance and MMSE score by the tertile of PAC.

As shown in Supplementary Table 1, the prevalence of low cognitive performance was 36.2% in the T1 group, 34.1% in the T2 group and 29.7% in the T3 group for total participants; 35.2% in the T1 group, 34.2% in the T2 group and 30.6% in the T3 group for apparently healthy participants; and in the diseased participants with and without taking medication, it was 36.5% in the T1 group, 33.1% in the T2 group and 30.4% in the T3 group; indicating that the prevalence of low cognitive performance gradually decreased with increasing aldosterone concentration. Analyses stratified by age, sex, and ethnic group were consistent with the main analysis.

As shown in Supplementary Table 2, average MMSE score of total participants were 26 in the T1 group, 26 in the T2 group and 27 in the T3 group; 27 in the T1 group, 27 in the T2 group and 28 in the T3 group for apparently healthy participants; and in the diseased participants with and without taking medication were 25 in the T1 group, 26 in the T2 group and 26 in the T3 group. With increasing aldosterone concentration, the MMSE score gradually increased. Analyses stratified by age, sex, and ethnic group were consistent with the main analysis.

Various sub-group analysis showed largely consistent results with the main analysis as in Supplementary Tables 4–6.

## Discussion

We explored the relationship between PAC and cognitive performance in a relatively large multi-ethnic population using a cross-sectional design.

Main results encompass: (1) as PAC increases, MMSE score increases. The multivariable linear regression analysis showed that log-MMSE score were positively associated with log-PAC in total, apparently healthy adults, and in the diseased without taking medicine participants. (2) as PAC increases, presence of low cognitive performance descends progressively. In logistic regression, the highest third tertile of PAC group showed significantly lower odds for the presence of MCI in total, in the diseased without taking medicine participants.

Aldosterone is a mineralocorticoid hormone, which has the physiological role of promoting the reabsorption of sodium and water and the excretion of potassium in the distal renal tubule and collecting duct (30). Aldosterone affects neuronal function, synaptic plasticity, cognitive function, and emotion regulation by binding to MR, widely distributed in the brain, particularly in the hippocampus and prefrontal cortex closely related to cognitive function (6). Overexpression of MR promotes differentiation and survival of embryonic stem cell-derived neurons (6). Aldosterone, the prototypic MR agonist, promotes the proliferation and survival of newly-generated granule cells in the dentate gyrus of adrenalectomized rat (9). Similarly, forebrain MR overexpression improves memory ability, and reduces neuronal loss during cerebral ischaemia in mice (7). A growing number of studies have shown that PI3K/Akt/GSK3b signalling pathway is a key regulator of various biological processes, including neurogenesis and synaptic plasticity (31, 32). In another animal study aldosterone induced the phosphorylation of Akt and GSK-3b, and promoted the proliferation of hippocampal neural stem cells and improved cognitive dysfunction in aged mice after surgery (8). Groch et al. (33) proved that MR activation is beneficial to the consolidation of declarative memory during sleep. Rimmele et al. (15) showed that MR blockade can impair the memory function of young healthy men. Stimulation of the MR has been previously found to improve memory in young and elderly healthy individuals, as well as depressed patients (16, 17). Some animal studies have shown that overexpression of MR enhances memory and stimulating MR enhances longterm potentiation, while decreased expression of MR in the hippocampus can lead to spatial memory impairment and working memory deficits (7, 10). Verpillat et al. (34) also found an association between ALD synthase (CYP11B2), a key enzyme gene for ALD synthesis, and WMLs seen on cerebral magnetic resonance imaging (MRI). Activation of the renin–angiotensin system has also been suggested to be involved in cognitive impairment through possible contributors including oxidative stress, inflammation, platelet aggregation and vasoconstriction (35–42). In addition, the inhibition of the renin-angiotensin system by angiotensin-converting enzyme inhibitors, angiotensin receptor blockers may be beneficial in alleviating cognitive deficits (43–46). Therefore, there is a complex relationship between plasma aldosterone and cognitive function. The potential impact of elevated aldosterone levels on cognitive function may be mediated through diverse mechanisms, including but not limited to oxidative stress, inflammation, and dysfunction of the blood–brain barrier (35–42). The existing body of research on the relationship between aldosterone and cognitive function is inconclusive, with contradictory results from studies conducted to date. The results of the animal studies suggest that aldosterone may improve cognitive function by binding to MR or by inducing Akt and GSK-3b phosphorylation (8). The existing body of research is predominantly focused on disease populations, and the majority of these studies have been conducted on individuals with elevated aldosterone levels, which complicates the determination of its protective or detrimental effects on cognitive function. Further research and exploration are necessary to clarify this issue. The results of our study suggest that under physiological conditions, aldosterone is not harmful to the human body and may even have a beneficial effect by protecting cognitive function. Future studies should delve into the specific mechanisms by which aldosterone affects cognitive function and assess the efficacy and safety of various interventions. The necessity for additional longitudinal studies is evident in order to explore these relationships in depth and to develop effective interventions to slow the risk of cognitive decline.

Several strengths merit this study as follow: First, this is one of the few large-scale population-based epidemiological studies to explore the association of PAC and cognitive function. According to the cross-sectional study sample size estimation formula to calculate sample size is far less than our data included in the number of people. Second, multiple confounders have been adjusted in the study such as age, gender, BMI, waist circumference, ethnicity, education, region, occupation, marital status, current smokers, current drinkers, systolic pressure, diastolic pressure, NoSAS score, PSQI score, MET Minutes, SDS score, SAS score, glutamic oxalacetic transaminase, total cholesterol, triglyceride, fasting blood glucose, which were previously proven to be associated with PAC and cognitive function. Third, Subgroup analyses also were performed by age, gender, BMI, living region, ethnicity and education attainment status. Although we hypothesized that differences in urban/rural distribution and ethnic group, due to many factors such as diet, would affect the relationship between circulating aldosterone and cognitive function, we found that the results of the subgroup analyses were consistent with those of the main analysis, indicating that group assignment had little effect on circulating aldosterone and cognitive function. Fourth, in the patient population, because of the activation of RAAS system, plasma aldosterone is not the actual level of aldosterone, but the final state of the disease (47); the RAAS system will be interfered by drugs, so the interference of drugs and diseases in the disease population will not achieve ideal analysis results (48). So we did separate analyses for the diseased and healthy populations, and for the diseased population who were not taking the medication, the results were consistent with the results of the main analysis.

However, some limitations should also be considered, while explaining the data. First, we used the MMSE scale to assess cognitive function, which has lower sensitivity and specificity. However, the MMSE scale is currently one of the most commonly used scales, especially in developing countries (49). The MMSE scale has subjectivity, language and comprehension biases, which have an impact on the results. MMSE assessments were administered in the participants’ primary language to reduce cultural or linguistic bias. We arranged investigators who are good at minority languages in areas where ethnic minorities live. In the data analysis, we stratified the analysis by ethnicity, education level, and region to address this limitation. Second, we failed to consider sodium and potassium, important regulators of aldosterone, and volume status, which may have brought bias to the results. However, the area where the study population live in is characterized by high sodium and low potassium intake intake (50)and therefore can be generalized to similar populations. Our previous article also mentioned this (18). Third, cross-sectional design makes it difficult to define causality and directionality of the association between PAC and low cognitive performance. However, since studies in animal models have suggested a positive effect of aldosterone on cognitive function, a positive effect of aldosterone in humans can be speculated. Fourth, PAC was measured without strict clinical testing standards, and samples were drawn from community physical examinations, and results may fluctuate. Traditionally, the test was performed in the recumbent position. However, recent studies demonstrated a higher accuracy for detection of angiotensin II responsive forms of PA with the test performed in the sitting position (51), which should therefore be the preferred approach (52, 53).

In conclusion, the present study demonstrated a positive correlation between plasma aldosterone and cognitive functions in community dwellers, particularly in apparently healthy adults. However, further studies are required to consider the cross-sectional nature of the current study.

## Data Availability

The raw data supporting the conclusions of this article will be made available by the authors, without undue reservation.
