# Supplementary material for: Cross-sectional association between plasma aldosterone concentration and cognitive performance by mini-mental state examination in community dwellers
Source: Front Nutr. 2025 Feb 6;12:1519644. doi: 10.3389/fnut.2025.1519644 (PMC11841653; doi:10.3389/fnut.2025.1519644)
Supplement: Supplementary file 1 [file Table_1.docx]

| Supplementary Table 1. the prevalence of low cognitive performance by the tertile of PAC in total participants, apparently healthy and diseased participants. | | | | |
| --- | --- | --- | --- | --- |
| Total participants | T1 | T2 | T3 | P |
| Total | 800 (36.2) | 752 (34.1) | 655 (29.7) | <0.001 |
| Age groups |  |  |  |  |
| ≤ 44 years | 199 (9.0) | 172 (7.8) | 209 (9.5) | 0.003 |
| 45–59 years | 331 (15.0) | 324 (14.7) | 247 (11.2) |  |
| ≥60 years | 270 (12.2) | 256 (11.6) | 199 (9.0) |  |
| men | 357 (16.2) | 263 (11.9) | 211 (9.6) | <0.001 |
| women | 443 (20.1) | 489 (22.2) | 444 (20.1) |  |
| Han (n,%) | 204 (9.2) | 259 (11.7) | 284 (12.9) | <0.001 |
| Ethnic minority (n,%) | 596 (27.0) | 493 (22.3) | 371 (16.8) |  |
| apparently healthy participants | | | | |
| Total | 277 (35.2) | 269 (34.2) | 241 (30.6) | 0.233 |
| Age groups |  |  |  |  |
| ≤ 44 years | 130 (16.5) | 125 (15.9) | 141 (17.9) | 0.010 |
| 45–59 years | 102 (13.0) | 106 (13.5) | 81 (10.3) |  |
| ≥60 years | 45 (5.7) | 38 (4.8) | 19 (2.4) |  |
| men | 110 (14.0) | 82 (10.4) | 64 (8.1) | 0.004 |
| women | 167 (21.2) | 187 (23.8) | 177 (22.5) |  |
| Han (n,%) | 59 (7.5) | 76 (9.7) | 83 (10.5) | 0.004 |
| Ethnic minority (n,%) | 218 (27.7) | 193 (24.5) | 158 (20.1) |  |
| diseased participants with and without taking medication | | | | |
| Total | 518 (36.5) | 470 (33.1) | 432 (30.4) | 0.013 |
| Age groups |  |  |  |  |
| ≤ 44 years | 70 (4.9) | 52 (3.7) | 62 (4.4) | 0.379 |
| 45–59 years | 225 (15.8) | 215 (15.1) | 173 (12.2) |  |
| ≥60 years | 223 (15.7) | 203 (14.3) | 197 (13.9) |  |
| men | 245 (17.3) | 175 (12.3) | 155 (10.9) | <0.001 |
| women | 273 (19.2) | 295 (20.8) | 277 (19.5) |  |
| Han (n,%) | 142 (10.0) | 177 (12.5) | 210 (14.8) | <0.001 |
| Ethnic minority (n,%) | 376 (26.5) | 293 (20.6) | 222 (15.6) |  |
|  | | | | |

| Supplementary Table 2. MMSE score by the tertile of PAC in total participants, apparently healthy and diseased participants. | | | | |
| --- | --- | --- | --- | --- |
| Total participants | T1 | T2 | T3 | P |
| Total | 26 (23, 28) | 26 (23, 28) | 27 (24, 29) | <0.001 |
| ≤ 44 years | 27 (25, 29) | 27 (25, 29) | 28 (26, 29) | <0.001 |
| 45–59 years | 25 (22, 28) | 26 (23, 28) | 27 (24, 29) |  |
| ≥60 years | 23 (20, 26) | 24 (20, 26) | 24 (21, 27) |  |
| men | 26 (23, 29) | 27 (24, 29) | 27 (25, 29) | <0.001 |
| women | 25 (22, 28) | 26 (22, 28) | 27 (24, 29) |  |
| Han | 27 (24, 29) | 27 (24, 29) | 27 (25, 29) | <0.001 |
| Ethnic minority | 25 (22, 28) | 26 (22, 28) | 26 (24, 29) |  |
| apparently healthy participants | | | | |
| Total | 27(24,29) | 27(24,29) | 28(25,29) | 0.644 |
| ≤ 44 years | 28(25,29) | 28(25,29) | 28(26,29) | <0.001 |
| 45–59 years | 26(23,28) | 26(23,28) | 27(24,29) |  |
| ≥60 years | 24(20,26) | 24(21,27) | 24(21,27) |  |
| men | 27(24,29) | 27(24,29) | 28(25,29) | <0.001 |
| women | 27(23,29) | 27(24,29) | 28(25,29) |  |
| Han | 27(25,29) | 27(25,29) | 28(26,29) | <0.001 |
| Ethnic minority | 26(23,29) | 26(23,29) | 27(24,29) |  |
| diseased participants with and without taking medication | | | | |
| Total | 25(22,28) | 26(22,28) | 26(23,29) | <0.001 |
| ≤ 44 years | 27(24,29) | 27(25,29) | 28(26,29) | <0.001 |
| 45–59 years | 25(22,28) | 26(23,28) | 26(24,29) |  |
| ≥60 years | 23(20,26) | 24(20,26) | 24(21,27) |  |
| men | 26(23,28) | 26(23,28) | 27(24,29) | <0.001 |
| women | 24(21,27) | 25(21,27) | 26(22,28) |  |
| Han | 26(23,28) | 26(23,28) | 27(24,29) | <0.001 |
| Ethnic minority | 25(21,27) | 25(21,28) | 26(23,28) |  |
|  | | | | |

| Supplementary Table 3. Univariate linear regression analysis between log plasma aldosterone concentration and log MMSE score in total population (B, 95%CI, P). | | | |
| --- | --- | --- | --- |
|  | B（95%CI）P | tolerance | VIF |
| logPAC | 0.04 (0.03, 0.04), <0.001 | 0.894 | 1.118 |
| Sex | -0.02 (-0.02, -0.01), <0.001 | 0.418 | 2.394 |
| Age | 0 (0, 0), 0 | 0.463 | 2.158 |
| Body mass index | 0 (0, 0), <0.001 | 0.253 | 3.959 |
| Waist circumference, cm | 0 (0, 0), <0.001 | 0.253 | 3.950 |
| Ethnicity | -0.02 (-0.02, -0.01), <0.001 | 0.836 | 1.197 |
| Education | 0.04 (0.04, 0.04), 0 | 0.686 | 1.457 |
| Region | -0.02 (-0.02, -0.02), <0.001 | 0.722 | 1.386 |
| Occupation | -0.04 (-0.04, -0.04), 0 | 0.672 | 1.488 |
| Marital status | -0.01 (-0.01, 0), 0.042 | 0.891 | 1.122 |
| Current smokers | 0.01 (0, 0.01), <0.001 | 0.823 | 1.215 |
| Current drinkers | -0.02 (-0.02, -0.02), <0.001 | 0.639 | 1.565 |
| Systolic pressure | 0 (0, 0), <0.001 | 0.465 | 2.151 |
| Diastolic pressure | 0 (0, 0), <0.001 | 0.502 | 1.994 |
| MET Minutes | 0 (0, 0), <0.001 | 0.911 | 1.097 |
| PSQI score | 0 (0, 0), <0.001 | 0.762 | 1.312 |
| NoSAS score | 0 (0, 0), <0.001 | 0.274 | 3.655 |
| SDS score | 0 (0, 0), <0.001 | 0.383 | 2.610 |
| SAS score | 0 (0, 0), <0.001 | 0.365 | 2.741 |
| Serum creatinine | 0 (0, 0), 0.169 | 0.956 | 1.046 |
| glutamic-pyruvic transaminase | 0 (0, 0), 0.520 | 0.843 | 1.186 |
| glutamic oxalacetic transaminase | 0 (0, 0), <0.001 | 0.840 | 1.190 |
| Total cholesterol | -0.01 (-0.01, 0), <0.001 | 0.868 | 1.152 |
| Triglyceride | 0 (0, 0.01), 0.048 | 0.845 | 1.184 |
| Fasting blood glucose | 0 (0, 0), <0.001 | 0.880 | 1.137 |

| Supplementary Table 4. Linear and logistic regression analysis for association of PAC with MMSE in total participants, stratified by region of residence, BMI, and ethnicity and educational status (B/OR, 95%CI, P). | | | |
| --- | --- | --- | --- |
|  | crude model | Model 1 | Model 2 |
| Linear regression analysis | | | |
| Stratification by living setting | |  |  |
| urban | 0.05 (0.04, 0.05), <0.001 | 0.01 (0, 0.01), 0.007 | 0.01 (0, 0.01), 0.012 |
| rural | 0.03 (0.02, 0.03), <0.001 | 0.01 (0, 0.01), 0.021 | 0.01 (0, 0.01), 0.016 |
| Stratification by BMI |  |  |  |
| <25kg/m2 | 0.04 (0.04, 0.05), <0.001 | 0.01 (0, 0.01), 0.048 | 0.01 (0, 0.01), 0.042 |
| 25-30 | 0.03 (0.03, 0.04), <0.001 | 0.01 (0, 0.02), 0.007 | 0.01 (0.01, 0.02), 0.003 |
| ≥30 | 0.03 (0.01, 0.04), <0.001 | -0.01 (0, 0.01), 0.890 | 0 (-0.01, 0.01), 0.982 |
| Ethnicity stratification |  |  |  |
| Han | 0.03 (0.02, 0.04), <0.001 | 0.01 (0, 0.01), 0.338 | 0.01 (0, 0.01), 0.156 |
| Ethnic minorities | 0.04 (0.03, 0.04), <0.001 | 0.01 (0, 0.01), 0.004 | 0.01 (0, 0.01), 0.007 |
| Stratification by education attainment status | |  |  |
| ≤Primary | -0.01 (-0.01, 0.01), 0.885 | 0.01 (-0.01, 0.01), 0.865 | -0.01 (-0.01, 0.01), 0.835 |
| Junior high | 0.02 (0.01, 0.03), <0.001 | 0.01 (0.01, 0.02), <0.001 | 0.01 (0.01, 0.02), <0.001 |
| ≥Senior high | 0.02 (0.01, 0.02), <0.001 | 0.01 (0, 0.01), <0.001 | 0.01 (0, 0.01), <0.001 |
| Logistic regression | | | |
| Stratification by BMI | |  |  |
| BMI<25 |  |  |  |
| T2 vs T1 | 0.90 (0.77, 1.06), 0.193 | 1.00 (0.84, 1.18), 0.954 | 0.98 (0.82, 1.17), 0.810 |
| T3 vs T1 | 0.81 (0.69, 0.95), 0.011 | 0.96 (0.80, 1.14), 0.614 | 0.93 (0.77, 1.12), 0.448 |
| BMI25-30 |  |  |  |
| T2 vs T1 | 0.93 (0.79, 1.10), 0.376 | 0.92 (0.77, 1.09), 0.313 | 0.89 (0.75, 1.07), 0.218 |
| T3 vs T1 | 0.75 (0.63, 0.90), 0.002 | 0.76 (0.63, 0.91), 0.004 | 0.73 (0.60, 0.88), 0.001 |
| BMI>30 |  |  |  |
| T2 vs T1 | 1.03 (0.81, 1.32), 0.794 | 1.02 (0.79, 1.32), 0.855 | 1.02 (0.79, 1.33), 0.876 |
| T3 vs T1 | 0.92 (0.71, 1.19), 0.534 | 0.92 (0.70,1.21), 0.563 | 0.86 (0.65, 1.15), 0.308 |
| Ethnicity stratification | |  |  |
| Han |  |  |  |
| T2 vs T1 | 0.99 (0.82, 1.19), 0.882 | 0.96 (0.79,1.16), 0.645 | 0.94 (0.77, 1.15), 0.554 |
| T3 vs T1 | 0.93 (0.78, 1.12), 0.461 | 0.93 (0.77, 1.13), 0.488 | 0.87 (0.71, 1.07), 0.190 |
| minority |  |  |  |
| T2 vs T1 | 1.01 (0.89, 1.15), 0.848 | 0.97 (0.85, 1.10), 0.630 | 0.95 (0.83, 1.09), 0.468 |
| T3 vs T1 | 0.89 (0.78, 1.02), 0.094 | 0.82 (0.71, 0.95), 0.007 | 0.79 (0.68, 0.92), 0.003 |
| Stratification by living setting | | | |
| urban |  |  |  |
| T2 vs T1 | 0.95 (0.80, 1.12), 0.525 | 0.99 (0.83, 1.18), 0.890 | 0.96 (0.80, 1.16), 0.678 |
| T3 vs T1 | 0.71 (0.60, 0.84), <0.001 | 0.72 (0.60, 0.87), <0.001 | 0.69 (0.57, 0.84), <0.001 |
| rural |  |  |  |
| T2 vs T1 | 0.91 (0.80, 1.04), 0.159 | 0.94 (0.82, 1.08), 0.388 | 0.93 (0.80, 1.07), 0.282 |
| T3 vs T1 | 0.84 (0.73, 0.96), 0.013 | 0.98 (0.84, 1.13), 0.744 | 0.95 (0.81, 1.11), 0.502 |
| Stratification by education attainment status | | | |
| ≤Primary |  |  |  |
| T2 vs T1 | 0.89 (0.75, 1.06), 0.188 | 0.85 (0.72, 1.02), 0.078 | 0.86 (0.72, 1.03), 0.110 |
| T3 vs T1 | 1.02 (0.85, 1.22), 0.872 | 1.01 (0.83, 1.22), 0.962 | 0.95 (0.78, 1.17), 0.641 |
| Junior high |  |  |  |
| T2 vs T1 | 0.80 (0.64, 0.98), 0.033 | 0.91 (0.73, 1.13), 0.383 | 0.92 (0.73, 1.15), 0.447 |
| T3 vs T1 | 0.60 (0.48, 0.76), <0.001 | 0.76 (0.59, 0.98), 0.033 | 0.74 (0.57, 0.96), 0.025 |
| ≥Senior high |  |  |  |
| T2 vs T1 | 1.04 (0.87, 1.24), 0.662 | 1.11 (0.93, 1.33), 0.247 | 1.04 (0.86, 1.26), 0.678 |
| T3 vs T1 | 0.67 (0.57, 0.79), <0.001 | 0.83 (0.69, 0.99), 0.039 | 0.80 (0.66, 0.96), 0.019 |
| Model 1 was adjusted for age, gender, BMI, waist circumference, ethnicity, education, region, occupation, and marital status. Model 2, adjusted for model 1+smokers, drinkers, SBP, DBP, PSQI score, MET Minutes, NoSAS score, SDS score, SAS score, total cholesterol, triglyceride, glutamic oxalacetic transaminase,and fasting blood glucose. | | | |

| Supplementary Table 5. Linear and logistic regression analysis for the association of PAC with MMSE in participants stratified by region of residence, BMI and ethnicity, and educational attainment status (B/OR, 95%CI, P) in people with no apparent disease. | | | |
| --- | --- | --- | --- |
|  | crude model | Model 1 | Model 2 |
| Linear regression analysis | | | |
| Stratification by living setting | |  |  |
| urban | 0.04 (0.03, 0.05), <0.001 | 0.01 (0, 0.01), 0.125 | 0.01 (-0.01, 0.01), 0.206 |
| rural | 0.02 (0.01, 0.03), <0.001 | 0.01 (0, 0.01), 0.083 | 0.01 (0, 0.01), 0.040 |
| Stratification by BMI | |  |  |
| <25kg/m2 | 0.03 (0.03, 0.04), <0.001 | 0.01 (0, 0.01), 0.180 | 0.01 (0, 0.01), 0.118 |
| 25-30 | 0.02 (0.01, 0.03), <0.001 | 0.01 (0, 0.02), 0.108 | 0.01 (0, 0.02), 0.045 |
| ≥30 | 0.03 (0.01, 0.05), 0.002 | 0.01 (-0.01, 0.03), 0.434 | 0.01 (-0.01, 0.03), 0.368 |
| Ethnicity stratification | |  |  |
| Han | 0.03 (0.02, 0.03), <0.001 | 0.01 (-0.01, 0.01), 0.336 | 0.01 (0, 0.01), 0.179 |
| Ethnic minorities | 0.03 (0.02, 0.03), <0.001 | 0.01 (0, 0.01), 0.057 | 0.01 (0.01, 0.02), 0.032 |
| Stratification by education attainment status | |  |  |
| ≤Primary | 0.01 (-0.01, 0.02), 0.835 | 0.01 (-0.01, 0.02), 0.735 | 0.01 (-0.01, 0.02), 0.652 |
| Junior high | 0.01 (0.01, 0.02), <0.001 | 0.01 (0.01, 0.02), 0.029 | 0.01 (0.01, 0.02), 0.031 |
| ≥Senior high | 0.01 (0.01, 0.02), <0.001 | 0.01 (0, 0.01), 0.047 | 0.01 (-0.01, 0.01), 0.088 |
| Logistic regression | | | |
| Stratification by BMI | |  |  |
| BMI<25 |  |  |  |
| T2 vs T1 | 1.08 (0.86, 1.35), 0.534 | 1.14 (0.90, 1.44), 0.275 | 1.10 (0.86, 1.40), 0.466 |
| T3 vs T1 | 0.92 (0.73, 1.15), 0.451 | 0.98 (0.76, 1.25), 0.854 | 0.94 (0.72, 1.22), 0.621 |
| BMI25-30 |  |  |  |
| T2 vs T1 | 0.84 (0.62, 1.14), 0.257 | 0.78 (0.57, 1.07), 0.126 | 0.79 (0.57, 1.09), 0.148 |
| T3 vs T1 | 0.86 (0.63, 1.18), 0.347 | 0.79 (0.57, 1.09), 0.156 | 0.76 (0.54, 1.08), 0.127 |
| BMI>30 |  |  |  |
| T2 vs T1 | 0.81 (0.46, 1.41), 0.448 | 0.83 (0.46, 1.49), 0.532 | 0.70 (0.38, 1.32), 0.270 |
| T3 vs T1 | 0.58 (0.30, 1.12), 0.106 | 0.58 (0.29, 1.17), 0.126 | 0.63 (0.30, 1.31), 0.213 |
| Ethnicity stratification | |  |  |
| Han |  |  |  |
| T2 vs T1 | 1.07 (0.75, 1.51), 0.714 | 1.01 (0.71, 1.44), 0.961 | 0.93 (0.64, 1.36), 0.716 |
| T3 vs T1 | 0.99 (0.70, 1.38), 0.930 | 0.93 (0.65, 1.34), 0.712 | 0.86 (0.59, 1.26), 0.444 |
| minority |  |  |  |
| T2 vs T1 | 1.01 (0.82, 1.24), 0.930 | 0.96 (0.78, 1.18), 0.676 | 0.93 (0.75, 1.16), 0.527 |
| T3 vs T1 | 0.96 (0.78, 1.19), 0.710 | 0.85 (0.68, 1.06), 0.146 | 0.81 (0.64, 1.02), 0.077 |
| Stratification by living setting | | | |
| urban |  |  |  |
| T2 vs T1 | 1.04 (0.79, 1.37), 0.788 | 1.06 (0.79, 1.41), 0.703 | 0.99 (0.73, 1.34), 0.944 |
| T3 vs T1 | 0.82 (0.62, 1.08), 0.159 | 0.80 (0.59, 1.07), 0.132 | 0.77 (0.56, 1.06), 0.111 |
| rural |  |  |  |
| T2 vs T1 | 0.90 (0.72, 1.12), 0.343 | 0.92 (0.73, 1.15), 0.459 | 0.89 (0.70, 1.14), 0.357 |
| T3 vs T1 | 0.83 (0.65, 1.05), 0.116 | 0.95 (0.74, 1.22), 0.694 | 0.93 (0.72, 1.21), 0.599 |
| Stratification by education attainment status | | | |
| ≤Primary |  |  |  |
| T2 vs T1 | 0.87 (0.63, 1.20), 0.396 | 0.83 (0.60, 1.17), 0.289 | 0.86 (0.61, 1.22), 0.408 |
| T3 vs T1 | 0.94 (0.65, 1.35), 0.726 | 0.95 (0.65, 1.39), 0.788 | 0.96 (0.64, 1.43), 0.833 |
| Junior high |  |  |  |
| T2 vs T1 | 0.80 (0.56, 1.13), 0.201 | 0.88 (0.62, 1.26), 0.499 | 0.92 (0.63, 1.34), 0.658 |
| T3 vs T1 | 0.56 (0.37, 0.84), 0.005 | 0.69 (0.45, 1.05), 0.084 | 0.65 (0.42, 1.03), 0.065 |
| ≥Senior high |  |  |  |
| T2 vs T1 | 1.09 (0.84, 1.41), 0.516 | 1.13 (0.87, 1.48), 0.362 | 1.03 (0.78, 1.36), 0.844 |
| T3 vs T1 | 0.81 (0.63, 1.05), 0.108 | 0.96 (0.73, 1.25), 0.752 | 0.97 (0.73, 1.28), 0.804 |
| Model 1 was adjusted for age, gender, BMI, waist circumference, ethnicity, education, region, occupation, and marital status. Model 2, adjusted for model 1+smokers, drinkers, SBP, DBP, PSQI score, MET Minutes, NoSAS score, SDS score, SAS score, total cholesterol, triglyceride, glutamic oxalacetic transaminase,and fasting blood glucose. | | | |

| Supplementary Table 6. Linear and logistic regression analysis for the association of PAC with MMSE in participants stratified by region of residence, BMI and ethnicity, and educational attainment status (B/OR, 95%CI, P) in people with apparent disease without taking medicine. | | | |
| --- | --- | --- | --- |
|  | crude model | Model 1 | Model 2 |
| Linear regression analysis | | | |
| Stratification by living setting | |  |  |
| urban | 0.05 (0.04, 0.06), <0.001 | 0.01 (-0.01, 0.02), 0.192 | 0.01 (-0.01, 0.02), 0.158 |
| rural | 0.03 (0.02, 0.04), <0.001 | 0.02 (0.01, 0.02), 0.001 | 0.02 (0.01, 0.02), 0.002 |
| Stratification by BMI | |  |  |
| <25kg/m2 | 0.06 (0.05, 0.07), <0.001 | 0.01 (0.01, 0.03), 0.014 | 0.01 (0.01, 0.03), 0.021 |
| 25-30 | 0.04 (0.02, 0.05), <0.001 | 0.01 (0, 0.02), 0.085 | 0.01 (0, 0.02), 0.065 |
| ≥30 | 0.04 (0.02, 0.05), <0.001 | 0.01 (-0.01, 0.02), 0.496 | 0.01 (-0.01, 0.02), 0.714 |
| Ethnicity stratification | |  |  |
| Han | 0.04 (0.03, 0.05), <0.001 | 0.01 (0, 0.02), 0.030 | 0.01 (0.01, 0.02), 0.016 |
| Ethnic minorities | 0.04 (0.02, 0.05), <0.001 | 0.01 (0, 0.02), 0.041 | 0.01 (-0.01, 0.02), 0.153 |
| Stratification by education attainment status | |  |  |
| ≤Primary | 0.01 (-0.01, 0.02), 0.533 | 0.01 (-0.01, 0.02), 0.401 | 0.01 (-0.01, 0.02), 0.782 |
| Junior high | 0.03 (0.02, 0.04), <0.001 | 0.02 (0.01, 0.03), <0.001 | 0.02 (0.01, 0.03), <0.001 |
| ≥Senior high | 0.02 (0.01, 0.03), <0.001 | 0.01 (0.01, 0.02), 0.010 | 0.01 (0.01, 0.02), 0.011 |
| Logistic regression | | | |
| Stratification by BMI | |  |  |
| BMI<25 |  |  |  |
| T2 vs T1 | 0.68 (0.52, 0.91), 0.008 | 0.73 (0.54, 0.99), 0.041 | 0.72 (0.53, 0.98), 0.034 |
| T3 vs T1 | 0.65 (0.49, 0.86), 0.003 | 0.72 (0.53, 0.99), 0.043 | 0.72 (0.52, 1.00), 0.052 |
| BMI25-30 |  |  |  |
| T2 vs T1 | 0.80 (0.61, 1.06), 0.125 | 0.75 (0.56, 1.01), 0.057 | 0.71 (0.52, 0.96), 0.026 |
| T3 vs T1 | 0.71 (0.53, 0.95), 0.021 | 0.69 (0.51, 0.95), 0.020 | 0.65 (0.47, 0.90), 0.010 |
| BMI>30 |  |  |  |
| T2 vs T1 | 0.78 (0.50, 1.21), 0.262 | 0.77 (0.49, 1.21), 0.260 | 0.86 (0.54, 1.37), 0.518 |
| T3 vs T1 | 0.82 (0.53, 1.26), 0.360 | 0.80 (0.50, 1.26), 0.330 | 0.84 (0.52, 1.34), 0.462 |
| Ethnicity stratification | |  |  |
| Han |  |  |  |
| T2 vs T1 | 0.72 (0.53, 0.97), 0.032 | 0.71 (0.52,0.97), 0.030 | 0.68 (0.49, 0.94), 0.021 |
| T3 vs T1 | 0.70 (0.52, 0.94), 0.018 | 0.72 (0.53, 0.99), 0.044 | 0.68 (0.49, 0.94), 0.021 |
| minority |  |  |  |
| T2 vs T1 | 0.87 (0.69, 1.09), 0.221 | 0.77 (0.61, 0.98), 0.035 | 0.77 (0.60, 0.98), 0.035 |
| T3 vs T1 | 0.86 (0.68, 1.10), 0.234 | 0.73 (0.56, 0.94), 0.016 | 0.73 (0.56, 0.95), 0.021 |
| Stratification by living setting | | | |
| urban |  |  |  |
| T2 vs T1 | 0.92 (0.68, 1.23), 0.561 | 0.96 (0.70, 1.31), 0.794 | 0.96 (0.70, 1.32), 0.791 |
| T3 vs T1 | 0.68 (0.51, 0.92), 0.011 | 0.70 (0.51, 0.96), 0.026 | 0.65 (0.47, 0.91), 0.011 |
| rural |  |  |  |
| T2 vs T1 | 0.64 (0.51, 0.81), <0.001 | 0.64 (0.50, 0.82),<0.001 | 0.61 (0.47, 0.78), <0.001 |
| T3 vs T1 | 0.67 (0.53, 0.86), 0.001 | 0.74 (0.58, 0.96), 0.024 | 0.73 (0.56, 0.96), 0.022 |
| Stratification by education attainment status | | | |
| ≤Primary |  |  |  |
| T2 vs T1 | 0.65 (0.48, 0.87), 0.003 | 0.57 (0.42, 0.77), <0.001 | 0.55 (0.40, 0.75), <0.001 |
| T3 vs T1 | 0.82 (0.61, 1.10), 0.185 | 0.78 (0.57, 1.07), 0.126 | 0.75 (0.54, 1.04), 0.084 |
| Junior high |  |  |  |
| T2 vs T1 | 0.63 (0.43, 0.91), 0.013 | 0.71 (0.48, 1.04), 0.080 | 0.71 (0.48, 1.05), 0.088 |
| T3 vs T1 | 0.61 (0.41, 0.90), 0.013 | 0.80 (0.53, 1.21), 0.289 | 0.80 (0.52, 1.23), 0.315 |
| ≥Senior high |  |  |  |
| T2 vs T1 | 0.93 (0.68, 1.27), 0.653 | 1.02 (0.73, 1.41), 0.930 | 0.99 (0.70, 1.39), 0.947 |
| T3 vs T1 | 0.55 (0.40, 0.75), <0.001 | 0.68 (0.49, 0.95), 0.025 | 0.63 (0.44, 0.89), 0.009 |
| Model 1 was adjusted for age, gender, BMI, waist circumference, ethnicity, education, region, occupation, and marital status. Model 2, adjusted for model 1+smokers, drinkers, SBP, DBP, PSQI score, MET Minutes, NoSAS score, SDS score, SAS score, total cholesterol, triglyceride, glutamic oxalacetic transaminase,and fasting blood glucose. | | | |
